# The cAMP-PKA signalling crosstalks with CWI and HOG-MAPK pathways in yeast cell response to osmotic and thermal stress

**DOI:** 10.15698/mic2024.03.818

**Published:** 2024-03-15

**Authors:** Fiorella Galello, Mariana Bermúdez-Moretti, María Clara Ortolá Martínez, Silvia Rossi, Paula Portela

**Affiliations:** 1 Universidad de Buenos Aires, Facultad de Ciencias Exactas y Naturales, Departamento de Química Biológica, Instituto de Química Biológica de la Facultad de Ciencias Exactas y Naturales-Consejo Nacional de Investigaciones Científicas y Técnicas (IQUIBICEN-CONICET). Buenos Aires, Argentina.

**Keywords:** Saccharomyces cerevisiae, stress, cAMP-PKA, CWI, HOG-MAPK, crosstalk

## Abstract

The yeast *Saccharomyces cerevisiae* is widely used in food and non-food industries. During industrial fermentation yeast strains are exposed to fluctuations in oxygen concentration, osmotic pressure, pH, ethanol concentration, nutrient availability and temperature. Fermentation performance depends on the ability of the yeast strains to adapt to these changes. Suboptimal conditions trigger responses to the external stimuli to allow homeostasis to be maintained. Stress-specific signalling pathways are activated to coordinate changes in transcription, translation, protein function, and metabolic fluxes while a transient arrest of growth and cell cycle progression occur. cAMP-PKA, HOG-MAPK and CWI signalling pathways are turned on during stress response. Comprehension of the mechanisms involved in the responses and in the adaptation to these stresses during fermentation is key to improving this industrial process. The scope of this review is to outline the advancement of knowledge about the cAMP-PKA signalling and the crosstalk of this pathway with the CWI and HOG-MAPK cascades in response to the environmental challenges heat and hyperosmotic stress.

## INTRODUCTION

Microorganisms have evolved responses that allow them to survive stressful challenges in constantly fluctuating external environments. Cellular responses to external stress are rapid, highly dynamic, plastic, complex, and involve the coordinated stimulation of many different pathways for the regulation of the gene expression at different levels [1]. The readjustments allow the equilibration of the effects of stress with the physiological requirements of the cell, guaranteeing that critical cell parameters are fine-tuned to ensure cell survival.

The yeast *Saccharomyces cerevisiae*, a single-celled microorganism used to produce alcoholic drinks and bread, has also been widely used as a genetic model system [2]. Yeast cells suffer the exposure to several types of stress as environmental conditions change, both in natural situations and during industrial processes. Both the damage caused by stress and the yeast response depend on the type and degree of stress and the developmental stage of the yeast at the time of the stimulus [3, 4]. Regardless of the type of stress exerted on the cells, a general stress response is induced. Therefore, when yeast cells are exposed to a mild stress, an increased tolerance to other stresses is achieved and restoration of cellular homeostasis is facilitated [5, 6]. When the buffering capacity fails to recover cellular homeostasis, cell death programs are stimulated to eliminate irreversibly damaged cells [7, 8].

*S. cerevisiae* has evolved mechanisms to sense, respond and adapt to these environmental changes. These mechanisms include several signal transduction pathways. Yeasts are pioneer organisms used to study in detail the feedback mechanisms, structure, organization and cellular responses through several signalling pathways to different stresses. The signalling pathways, usually conformed by kinase cascades, allow a tight control of the response to a specific signal [9–11]. The compartmentalization of intracellular effectors, via adaptors or anchor proteins, is critical to the temporal and spatial control of signal transduction. Although several types of stress have been studied in yeast, the complete stress-activated network and the principles that control signal integration remain incomplete [12–15].

When *S. cerevisiae* grows in optimal environmental and nutrient conditions, expression of growth-related genes is high and expression of genes involved in stress defense is low. One of the transduction pathways involved in the regulation of this balance is that of cAMP-protein kinase A (PKA) [4, 13, 14]. Unfavourable conditions turn off this pathway and, at the same time, stress-specific signalling networks are activated and allow coordinated changes at the level of transcription, translation, post-translational modifications, and metabolic fluxes [4]. This leads to an appropriate response to each stress situation.

The well-known cAMP-PKA pathway responds to external stimuli through the modulation of the second messenger cAMP, which activates the PKA [16, 17]. *S. cerevisiae* PKA is a tetrameric holoenzyme consisting of a regulatory subunit (Bcy1) dimer and two catalytic subunits (Tpk1, Tpk2 and Tpk3). A single gene *BCY1* encodes the regulatory subunit, while there are three genes, *TPK1*, *TPK2* and *TPK3,* encoding the catalytic subunits [18]. When PKA is in its inactive state, the Bcy1 dimer is bound to two catalytic subunits (Tpk). In response to different stimuli, cAMP increases, and the Bcy1 dimer undergoes conformational changes that promote the catalytic subunits release, which phosphorylate their target substrates [19–21]. The output is a wide variety of specific responses. The cAMP-PKA signalling pathway in *S. cerevisiae* has also been associated with the regulation of ageing, budding, actin repolarization, glycogen accumulation, stress resistance, sporulation, pseudohyphal differentiation, fermentative growth, stationary phase entry, and transcriptional regulation in response to different stimuli [19–23].

This article reviews the current state of knowledge of the cAMP-PKA pathway involvement and the crosstalk with the CWI and HOG-MAPK cascades in the response to environmental challenges focusing on heat and hyperosmotic stress in *S. cerevisiae.*

## ROLE OF THE cAMP-PKA PATHWAY IN THE CELLULAR RESPONSE TO STRESS

Under stressful growth conditions, *S. cerevisiae* activates both transcriptional and physiological protective mechanisms. The stressed yeast cells activate specific transcription changes; thus, the expression of specialized genes is modulated to address the particular stress condition [24, 25].

Genomic expression and global phosphoproteome studies shed light on the modulation of genes and protein phosphorylation involved in carbohydrate metabolism, protein folding degradation and processing in response to environmental stress, nutrient starvation and carbon source [26–31]. The expression patterns of these genes during the adaptation to diverse stressful environments were termed as “Environmental Stress Response” (ESR) [3, 32, 33]. Actively growing cells are more sensitive to stress than quiescent cells [34].

In *S. cerevisiae*, one of the central controls of the ESR is the cAMP-PKA signalling, which transduces the changes in environmental conditions. The cAMP-PKA pathway is repressed in response to stress, and other signalling pathways are activated to coordinate the transcriptional and translational modifications as well as the changes in the metabolic flux along with cell cycle arrest. The importance of these pathways in the adaptive response to stress is evident in mutants with hyperactive cAMP-PKA pathway. These mutants show very low tolerance to stress, decreased viability in stationary phase, and no trehalose and glycogen accumulation. On the other hand, mutations that decrease the PKA activity result in phenotypes with high tolerance to stress, increased accumulation of glycogen and trehalose, even in actively proliferating cells [14, 35]. Furthermore, under many conditions, cAMP levels are high, resulting in the activation of PKA and accordingly the fermentative growth is promoted. As glucose is depleted, yeast reduces cAMP levels and switches to ethanol-oxidative metabolism. Once cells reach the stationary phase, cAMP levels remain low. On the other hand, under stressful conditions, the cAMP levels decrease and, thus, the low PKA activity results in the inhibition of programs of genes that regulate growth and, at the same time, in the upregulation of stress responsive genes [4, 15, 36]. These findings suggest that fluctuations in cAMP levels play a significant role in the regulation of growth, fitness, and stress adaptability.

The promoters of most genes induced by the ESR contain the STRE element, the binding site for the non-redundant Msn2 and Msn4 transcription factors. Regulation by one or the other of these transcriptional factors depends on the promoter context and the type of stress. PKA regulates the nuclear localisation and therefore the activity of Msn2/4. High PKA activity induces Msn2/4 phosphorylation, which maintains their cytoplasmic localisation and thus suppresses their activity. On the contrary, when the PKA activity is low, Msn2 localisation is predominantly nuclear and it is active [37–41]. The subcellular localisation of Msn2 in yeast is dynamic, occurring in bursts in response to rapid pulses of PKA activity [42–45]. Later results indicate that the phosphorylation of Msn2 by Tpk1 and Tpk3 isoforms leads to the inhibition of its activity, while Tpk2 seems to function as a partial activator of Msn2 [46].

### Osmotic stress and PKA

An increased extracellular osmolarity generates hyperosmotic stress. The addition of high concentrations of salts as NaCl or KCl to *S. cerevisiae* cell cultures generates osmotic and ionic stress [47]. During the response to these stresses, the concentration gradient promotes ion movement into the cell and the diffusion of water out of the cell to balance the osmotic pressure across the plasma membrane. The result is the sudden reduction in cellular volume and the cell cycle arrest. The cell responds rapidly by increasing the intracellular glycerol concentration, which causes water to re-enter the cell. So, the original cell volume and turgor are restored [48].

*S. cerevisiae* responds to osmotic stress through two main mechanisms. One of them involves the osmolyte exporter Fps1. This channel remains closed upon hyperosmotic conditions preventing glycerol from exiting the cell [49, 50]. The other mechanism involves the bona fide sensors, Sln1 and Sho1, that control the HOG-MAPK (High Osmolarity Glycerol-Mitogen Activated Protein Kinase) pathway [51, 52]. The MAPK of this pathway, Hog1, acts on cytoplasmic and nuclear targets to modify cellular metabolism to increase glycerol synthesis [53–55]. The HOG pathway includes three sequentially acting protein kinases named MAPK, MAPK kinase (MAPKK, MAP2K), and MAPKK kinase (MAPKKK, MAP3K) [48, 55]. There are two sensors that conform two signalling branches, Sho1 and Sln1, which detect osmostress independently and activate MAP3Ks. Both Sho1 and Sln1 activate the Ste11-Pbs2-Hog1 MAPK cascade and the Ssk2/Ssk22-Pbs2-Hog1 MAPK cascade, respectively. Pbs2 MAP2K is activated by phosphorylation [56–60]. The activated Pbs2 can phosphorylate Hog1, and phosphorylated Hog1 translocates to the nucleus [61]. Hog1 allows the adaptive responses to osmostress of yeast cells, inducing the modulation of intracellular glycerol levels, metabolism, ion transporters, and translation. In addition, Hog1 regulates gene expression of osmostress-responsive genes [55, 57, 62]. The severity of the stress modulates Hog1 activation, which is negatively regulated by protein phosphatases [55].

The transcriptional regulation of Hog1 target genes occurs through diverse mechanisms, involving physical interaction with transcription factors as Msn2/4, Hot1, Tup1-Ssn6 and other transcriptional regulatory proteins [63, 64]. Hog1 can also bind to the coding regions of stress-responsive genes and activates by phosphorylation the transcription elongation factors Spt4 and Spt5 [65]. Recently, it has been proposed another mechanism by which Hog1 regulates the expression of genes by modulating the activity of the 5'-3' exoribonuclease Xrn1 [66]. Finally, it has been described the association of Hog1 to promoter regions of stress-responsive genes to facilitate the recruitment of RNA Pol II and the chromatin remodelling complexes SWI/SNF or INO80, allowing gene activation or repression respectively [67–69].

PKA also regulates gene expression under osmotic stress in addition to doing so through the HOG pathway. It was demonstrated that PKA activity levels affect osmotolerance and modulate the expression of osmo-responsive genes in *S. cerevisiae* [70]. However, Hohmann *et al.* proposed that PKA mediates ESR not only upon osmostress but also under several other stress conditions such as high ethanol levels, thermal stress, oxidative stress, or nutrient starvation. Therefore, the regulation by PKA may not be exclusively linked to osmotic changes [71]. However, other results indicate that the regulation of ESR genes depends on the modulation of Msn2/4 activity by nuclear translocation [40, 44, 72], phosphorylation and degradation [40, 73, 74]. At some of these regulation levels, the signalling pathways cAMP-PKA and HOG-MAPK have important roles [38, 39, 41, 43, 74–77].

In stress conditions, several protein kinases regulate gene expression through the binding to chromatin in either promoters or coding regions and through phosphorylation of histones, transcription factors, chromatin remodeler complexes and transcription machinery. PKA [78, 79] and Hog1 [67, 80, 81] have been described as chromatin associated kinases. Baccarini *et al.* [82] demonstrated the importance of PKA chromatin association in the regulation of osmostress-responsive genes. During osmotic stress Tpk1 accumulates in the nucleus, while Tpk2 and Bcy1 maintain the nuclear-cytoplasmic localisation. The authors also demonstrated that in response to osmotic stress, PKA subunits bind to different gene regions of osmo-inducible genes. Both Tpk1 and Tpk2 subunits are recruited to the coding regions, and Tpk2 is also bound to the promoters of ribosomal protein genes. Tpk1 and Tpk2 mutant versions without catalytic activity do not bind the genes analysed so far. A mutant strain containing a deletion of *BCY1* gene which has a deregulated PKA activity, shows an increased Tpk1 but not Tpk2 recruitment. Furthermore, this mutant strain shows a higher binding rate of the remodelling complexes SWI/SNF and INO80, and also, an upregulated gene expression under hyperosmotic conditions. When PKA binds to chromatin, it can phosphorylate nearby substrates, which could be transcription factors or chromatin remodelers. Thus, there are distinct mechanisms by which Tpk1 and Tpk2 catalytic subunits bind to chromatin, resulting in increased specificity in response to osmotic stress.

### Crosstalk between cAMP-PKA and HOG-MAPK pathways during osmostress

Diverse stimuli interact and cross-activate different signalling pathways in the cells. In *S. cerevisiae,* there are several examples in which multiple signalling pathways function in a coordinated manner to respond to stimuli. The specific response to an input signal of the different MAPK pathways described [72, 83, 84], which share several components, requires both insulation mechanisms and the coordinated communication among them. For instance, high osmolarity glycerol (HOG MAPK) pathways, mating programs (pMAPK) and filamentous growth (fgMAPK) can maintain the fidelity of the responses by restricting signalling complexes to discrete subcellular compartments and by switching on mechanisms to avoid crosstalk between MAPK cascades. In fact, in *hog1*Δ cells subjected to high osmolarity conditions, the pMAPK pathway is activated in contrast to wild-type cells. This way, the activation of the mating pathway is inhibited by Hog1 activity [85, 86]. In response to osmotic stress, Hog1 also prevents the activation of the fgMAPK pathway by inhibiting the MAPKKK Ste11 of the SHO1 branch [55, 86].

Among the MAPK pathways present in *S. cerevisiae*, the Cell Wall Integrity (CWI) pathway is key to overcoming the cell wall damage caused by stressful conditions as chemical agents affecting cell wall biogenesis [87]. Several other stressors such as heat stress, ethanol, hypo- and hyperosmotic shock, oxidative stress, among others that affect secondary cell wall structure also activate CWI signalling [88, 89]. A more detailed description of this signalling cascade is developed in the following section. Another example of crosstalk occurs during polarised growth in mating and in pseudohyphal development, where the activity of fMAPK and pMAPK pathways in coordination with the CWI to allow cell wall remodelling are required [90–92].

The relationship between the HOG and cAMP-PKA pathways in *S. cerevisiae* has also been described. A high-throughput approach, using a typical Msn2/4-regulated reporter gene, was employed across different genetic backgrounds, including single-knockout and double-knockout, and various stress conditions. By measuring reporter activity and analysing phenotypes, as well as the nuclear localisation of Msn2, Gutin, J. *et al.* clear up the signalling and transcriptional networks that regulate Msn2/4 activity. The results show new inter-pathway interactions between the cAMP-PKA and HOG MAPK pathways in response to heat shock, osmotic stress, and redox stress [93].

In addition, our own unpublished results suggest that the HOG-MAPK and cAMP-PKA pathways interact during osmotic stress. PKA catalytic isoforms, Tpk1 and Tpk2, show different roles in the adaptive response to osmotic stress. The lack of *TPK2* gene improves the defective cell growth of *HOG1*-deficient strains under osmotic stress. Also, there is a negative correlation between *TPK2* expression and processes such as growth rate during the exponential phase, glucose consumption, and trehalose accumulation in a *hog1Δ* strain under osmotic stress conditions. In contrast to *TPK2*, *TPK1* expression has a smaller effect on restoring the defective cellular response under osmotic stress in cells with an inactive HOG-MAPK pathway.

**Figure 1:**
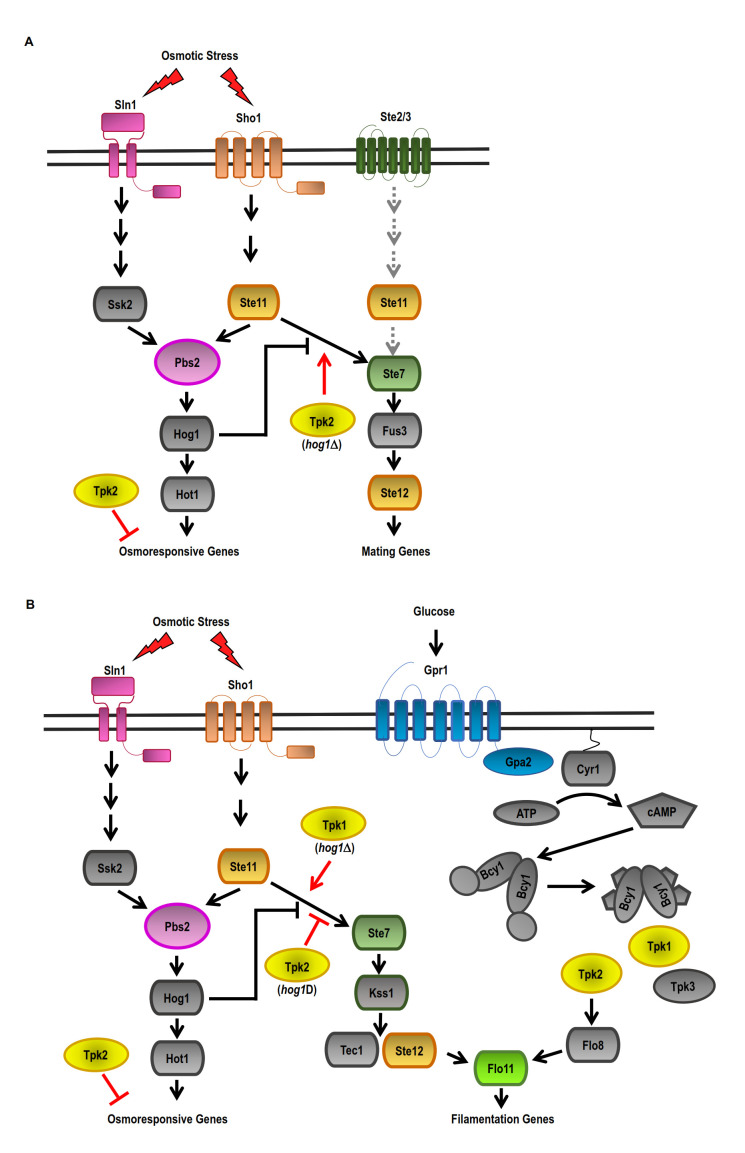
FIGURE 1: Model of the crosstalk between cAMP-PKA and HOG pathways in response to osmotic stress. **(A)** Two independent osmosensing mechanisms, the Sln1 and Sho1 branches, lead to the activation of specific kinases Ssk2/22 and Ste11 (MAPKKK) that converge on the common MAPKK Pbs2, which activates the Hog1 MAPK. In response to osmotic stress, Hog1 inhibits the crosstalk with the pheromone MAPK pathway (pMAPK) via inhibition of the MAPKKK Ste11 of the SHO1 branch. Upon *HOG1* deletion (*hog1*Δ), the crosstalk between the HOG-MAPK and pMAPK pathways can be positively regulated by Tpk2. **(B)** In response to osmotic stress, Hog1 inhibits the crosstalk of the filamentous MAPK pathway (fgMAPK) by inhibiting the MAPKKK Ste11 of the SHO1 branch. In a *hog1*Δ background, Tpk2 negatively regulates the crosstalk between the HOG-MAPK and fgMAPK pathways, while Tpk1 positively regulates the crosstalk between the HOG-MAPK and fgMAPK pathways in response to osmotic stress. In the presence of glucose, Tpk2 positively regulates invasive growth.

Yeast mating is initiated by pheromone stimulation, which triggers a MAPK cascade made up of Ste11, Ste7, and finally the MAPKs Fus3 and Kss1. During mating, the cell cycle is arrested by high concentrations of pheromone, and polarized cell growth is induced to form cellular projections called “shmoo” morphology [94–96]. Yeast cells lacking *HOG1* gene show a “shmoo-like” morphology in response to osmotic stress due to the crosstalk between the HOG-MAPK and pMAPK pathways [86, 97]. Our findings showed that the PKA catalytic subunits Tpk2 and, to a lesser extent, Tpk1, can reduce the crosstalk between the pheromone MAPK pathway and HOG-MAPK in a *hog1Δ* strain (unpublished results) (**Figure 1A**). In *S. cerevisiae,* filamentous growth is regulated by nutrient availability and the conserved filamentous MAPK pathway (fgMAPK) [97]. However, the cAMP-PKA pathway activation is also required for this type of growth. Invasive growth is positively regulated by the cAMP-PKA pathway in response to glucose sensing and by the fgMAPK pathway in response to nitrogen-free medium [98, 99]. Deletion of *TPK2*, but not *TPK1*, prevents filamentous growth. In addition, deletion of *TPK3* produces hyperfilamentous growth, indicating that Tpk3 is an inhibitor of this growth [100, 101]. In hyperosmotic conditions, a *hog1Δ* strain exhibits invasive growth which is regulated by a crosstalk between the HOG1-MAPK and fgMAPK pathways [86]. The role of Tpk2 subunit on the crosstalk between the fgMAPK and HOG MAPK pathways was analysed in a strain with deficiencies in the expression of *FLO8* gene. This strain is prevented from pseudohyphal growth [99]. In a *hog1Δ* mutant and under high osmolarity, the Tpk1 isoform acts as a positive regulator, whereas the Tpk2 isoform serves as a negative regulator in the crosstalk between the fgMAPK and HOG-MAPK pathways (**Figure 1B**). The Tpk1 and Tpk2 isoforms have distinct functions in cell morphology and invasive growth under osmotic stress conditions.

Thus, Hog1 positively acts in an adaptive response to osmotic stress, while PKA negatively acts via inactivation of Tpk1 and, for the most part, Tpk2 isoforms functions. The specific role that each Tpk plays in the cellular response to osmotic stress remains obscure. Evidence suggests that *TPK1* or *TPK2* deletions do not affect the phosphorylation of Hog1 induced by osmotic stress or Hog1 nuclear accumulation. Thus, cAMP-PKA signalling is controlling the effectors that are downstream targets of the HOG-MAPK pathway. In this sense, evidence suggests that PKA and HOG pathway crosstalk may occur at the nuclear level. Under osmotic stress conditions, the expression of *TPK2* and *HOG1* genes affects the kinetics of the binding of Tpk2 and Hog1 to the chromatin. Also, Tpk2 and Hog1 affect the association of Snf2 (SWI/SNF complex) and Msn2 with the promoters of osmosis-responsive genes (unpublished results).

Overall, when the cells fail to activate the HOG-MAPK pathway, crosstalk between signalling pathways allows a coordinated response where downregulation of the cAMP-PKA pathway produces a better adaptive response to osmo stress. The lack of the *HOG1* gene results in the inactivation of Tpk2 activity, which results in insulation between MAPK pathways. Changes in the dynamics of Tpk2 association with chromatin and, in turn, changes in gene expression regulation in reaction to osmotic stress would be part of this adaptive mechanism. The next challenge is to understand the mechanism that controls the specific inactivation of Tpk2 isoform under osmotic stress.

### Thermal stress and PKA

At suboptimal temperatures, different protective mechanisms are activated in *S. cerevisiae*, including a transcriptional gene expression program known as the Heat Shock Response (HSR) [28, 102]. During this response the expression of genes involved in protein biosynthesis pathways is downregulated and heat-shock proteins genes are upregulated [103]. The HSR is also activated by other stresses such as heavy metals exposure, oxidative stress and alterations in protein conformation [104]. In addition, yeast cells modify the membrane composition and their metabolism [105]. The upregulation of heat-shock genes is driven by the transcription factor Hsf1 (Heat Shock Factor) [104]. This factor is inactive under non-stress conditions but active when heat-induced misfolded proteins are accumulated in the cell. All these changes induced by thermal stress ensure the maintenance of proteostasis and metabolism [106]. In *S. cerevisiae,* the above mentioned Msn2/4 are a second kind of transcription factors that regulate the heat-shock gene expression. The expression regulation by Msn2/4 transcription factors is much more extensive than transcripts induced by heat shock, since it includes genes induced by other stresses in the general Environmental Stress Response. The shifts in the transcription levels of the HSR genes are the result of transcriptional changes and also differences in mRNA stability [107].

As it was mentioned before, PKA inhibits the Msn2/4 function, but in addition, other signal transduction pathways also regulate their activity in response to different environmental conditions, through factors as Mck1, Rim15, Yak1, Snf1, and Hog1 [46]. PKA activity is dispensable in the double deletion mutant strain of *MSN2* and *MSN4* genes. Therefore, the targets regulated by Msn2 and Msn4 stimulate genes that inhibit growth antagonizing the PKA dependent growth [37]. There is evidence that suggests that Yak1 kinase would fulfill this role [37, 75].

In response to heat shock, Msn2/4, like Hsf1, is hyperphosphorylated; however, this modification is inhibited by cAMP. Therefore, the hyperphosphorylation might not be mediated by PKA [74]. However, heat shock slightly decreases cAMP levels through the destabilization of Cdc25, activator of Ras1/2 and adenylyl cyclase [108]. Thus, cAMP-PKA could be the link between stimulus and response in HSR signalling although additional phosphorylation events may also act as regulating this response [108].

The assembling of the ribonucleoprotein (mRNP) composed of mRNAs and RNA-binding proteins (RBPs) is critical in the mRNA fate. During stress conditions, some mRNPs aggregate into larger complexes assembling membraneless organelles named RNP granules. There are many different types of cytoplasmic RNP granules; Stress Granules (SG) and Processing Bodies (PB) are two examples of them. Both types of granules participate in several aspects of mRNA metabolism as storage, localisation, translation and decay [109–111].

However, how different types of stress impact the formation of RNP granules is an unresolved question. PBs and SGs contain several groups of proteins as well as mRNAs, and these proteins participate in the biological activities of the granules. Among these proteins, different protein kinases and phosphatases have also been found associated with PBs [112–117]. PKA has a key role in the regulation of PBs and SGs assembling in response to glucose deprivation and stationary phase entry [116–118]. In addition, PKA regulates the assembly of PBs and SGs and protein translation upon heat stress in *S. cerevisiae*. It was shown that Tpk1, Tpk2 and Tpk3 isoforms have different roles in the assembling of SGs and PBs induced by thermal stress [119]. In conditions of moderate heat stress, Tpk3 aggregates and induces the assembly of proteins implicated in translation as eIF4G, Pab1 and eIF4E. However, these Tpk3 granules are neither PB nor SG. By contrast, upon severe heat stress the assembling of PBs and SGs containing both Tpk2 and Tpk3 and the 48S translation initiation complex are induced. Therefore, Tpk2 plays a positive role promoting mRNA translation and negative in the number and size of SGs and PBs. On the contrary, Tpk3 inhibits the assembling of SGs and PBs and appears to be involved in translational repression [119].

*TPK1* has no effect on the SGs and PBs evoked by heat shock. The localisation of Tpk2 is dependent on its kinase activity, but Tpk3 kinase activity is not necessary for its accumulation in cytoplasmic foci [120]. Each catalytic subunit isoform plays opposite roles in translational response to severe heat stress. However, heat stress does not affect its intrinsic Tpk kinase activity [119]. Global characterization of Tpk-associated protein complexes under heat stress includes tools such as affinity-purified complexes and mass spectrometry. These tools would allow the determination of a specific pattern of PKA substrate phosphorylation in response to heat stress.

### Crosstalk between cAMP-PKA and CWI pathways during heat stress

**Figure 2:**
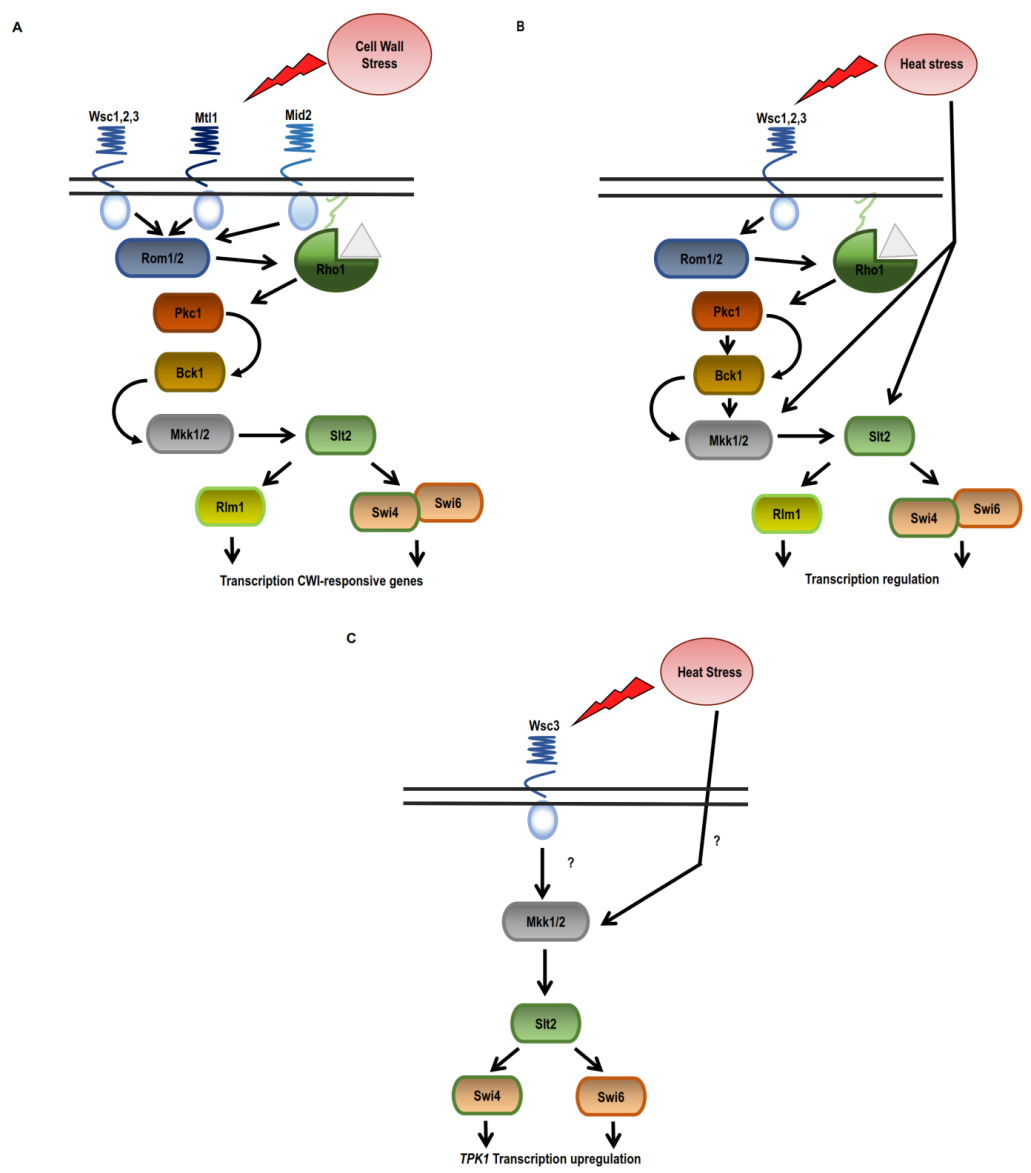
FIGURE 2: Crosstalk between cAMP-PKA and CWI pathway in response to heat stress. **(A)** Damage in the cell wall is sensed via Wsc1-3, Mtl1 and Mid2 sensors that trigger the CWI cascade conformed by Bck1, Mkk1/Mkk2, and Slt2 through Rho1 and the activation of Pkc1. **(B)** Upon heat stress, CWI pathway is activated by a different mechanism, regulating the kinases Mkk1,2 and Stl2 rather than Rho1 or Pkc1. Heat stress would be acting as a lateral input rather than operating in a linear “top-down” manner. **(C)** Tpk1 expression regulation during heat stress. Tpk1 upregulation depends on the Wsc3 membrane sensor Mkk1 and the transcription factor Swi4.

As mentioned before, high temperatures induce the activation of the HSR and the CWI pathways in yeast cells [120]. This environmental condition was described to activate the CWI MAPK cascade [87, 121]. Changes in plasma membrane composition in response to thermal stress were described [89]. There are different CWI sensors described, namely Wsc1–3, Mid2 and Mtl1 [122, 123], but how these sensors detect thermal stimuli is not fully understood and is controversial. Yeast strains carrying single deletion mutations, *wsc1Δ*, *wsc2Δ*, or *wsc3Δ,* are thermo-tolerant, while the respective double mutants are thermosensitive [124]. It was also described that upon heat shock, Wsc1, Wsc2, and Mid2 activate Rom2 promoting GTP loading of Rho1, and the consequent Pkc1 activation [125]. In cells lacking the sensors Mid2 or Wsc1-3, the HSR is activated, but the cells are sensitive to heat shock and autolytic [122, 123]. Therefore, it was proposed that the sensors have overlapping functions although they are also specific. Subsequently, it was also described that upon heat shock the Wsc receptors have an additive effect [122, 126]. Downstream of the membrane sensors (Wsc1-3, Mid2 and Mtl), the signal is amplified by a MAPK cascade [122, 123]. Through Rom1/Rom2 and the small G-protein Rho1, these sensors stimulate the downstream kinase Pkc1, which activates the MAPK cascade conformed by Bck1 and Mkk1/2. Finally, Mkk1/2 kinases activate the MAPK Slt2, and this kinase regulates the activity of Rlm1 and Swi4/6 transcription factors. The result is the regulation of genes involved in cell wall biogenesis [87, 103] (**Figure 2**).

CWI pathway is usually activated as a hierarchic top-down cascade; however, some stress stimuli can regulate this pathway at different steps of the cascade downstream of Rho1. Some reports show that the activation of Slt2, the last kinase of the cascade, may come from another step in this MAPK cascade. Indeed, upon thermal stress, Slt2 is phosphorylated in a CWI sensor independent manner [127–129]. Thus, heat shock can activate the CWI signalling at the Mkk1/2 and/or Stl2 cascade steps [127] (**Figure 2**).

The crosstalk between the CWI and PKA signalling pathways was also studied. Yeast cells deficient in *IRA2* (GTPase-activating protein that negatively regulates RAS) are not thermotolerant; however, the deletion of *WSC1* reverses this phenotype. The authors proposed that Wsc1 negatively regulates targets of RAS1/2. Indeed, the deletion of Ras2 rescues the heat shock sensitivity of a *wsc1Δ* strain. Thus, Ras1/2 and Wsc1 have opposing effects on any downstream target [124]. Later, it was demonstrated that the Wsc1 sensor also contributes to the crosstalk between CWI with the cAMP-PKA pathway at the level of Slt2. It was described that Sdp1, a phosphatase that negatively regulates Slt2, is transcriptionally regulated by the transcription factors Msn2/Msn4 [130].

**Figure 3:**
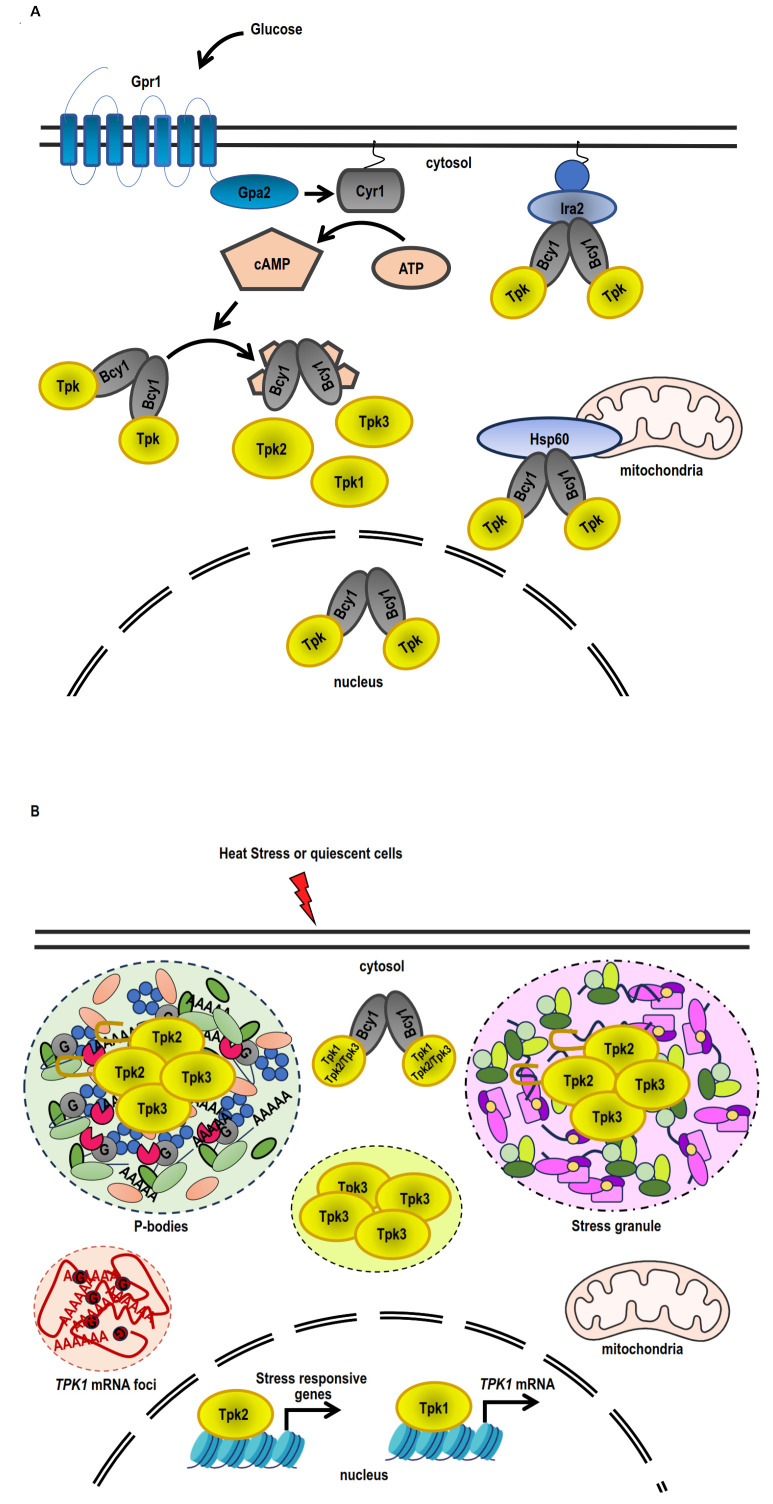
FIGURE 3: Differential PKA subunit regulation in response to stress and quiescence. **(A)** Under conditions of exponential growth on glucose, Bcy1 and Tpk2 are mostly localised within the nucleus, while Tpk1 and Tpk3 are found in both the nuclear and cytoplasmic compartments. The PKA holoenzyme (Bcy1_2_-Tpks_2_) is tethered to the membrane-attached Ras complex by Ira2, whereas the association of the holoenzyme to the mitochondria is facilitated by Hsp60. Adding glucose causes the Ras complex and the heterotrimeric G protein-coupled Gpr1 receptor to stimulate adenylate cyclase (Cyr1), which increases the amount of cAMP inside the cell. An increase in cAMP levels triggers the dissociation of the PKA holoenzyme into a dimer of Bcy1 and two Tpk catalytic subunits, which are able to phosphorylate substrates. **(B)** Tpk2 and Tpk3 localise with P-bodies and/or stress granules in the cytoplasmic foci during quiescence or heat stress. The prion-like domain in N-terminus of Tpk2 facilitates its localisation into P-bodies. Tpk3 localises to foci in the cytoplasm that are distinct from conventional SGs or PBs. The locations of Tpk1 and Bcy1 in the cytoplasm are diffuse. Tpk1 and Tpk2 interact with stress-responsive genes, such as the *TPK1* promoter, inside the nucleus. Heat stress triggers an increase in *TPK1* mRNA expression, which subsequently accumulates in cytoplasmic foci. Foci sequestration of *TPK1* mRNA inhibits translation entry.

CWI signalling also plays a role in the regulation of *TPK1* expression during heat shock [131]. Previously, it was described that Tpk1 protein levels remain unchanged upon heat shock although *TPK1* mRNA is upregulated and the half-life of *TPK1* mRNA increases. This mRNA is localised in cytoplasmic foci that are not disassembled after cycloheximide treatment (**Figure 3B**). The fact that these foci are resistant to cycloheximide treatment and results from the polysome profiling analysis indicate that *TPK1* mRNA is impaired for entry into translation. Therefore, in response to heat shock, Tpk1 levels are regulated by a post-transcriptional mechanism that involves the assembling of *TPK1* mRNA granules that are translationally silent. In this regulation, the CWI components Wsc3 sensor and Mkk1 are necessary for *TPK1* expression upon heat-shock. However, the participation of Slt2 is not absolutely defined. The *TPK1* mRNA foci evoked upon thermal stress depend on Wsc3 but not on the other sensors. The levels of Tpk1 protein are lower in a *wsc3Δ* mutant than in a wild-type strain, and consequently PKA levels are also lower, as was demonstrated by phenotype analysis. Regarding the participation of the transcription factors Swi4 and Swi6, it was published that apparently only Swi4 seems to be necessary for the regulation of *TPK1* expression [131]. Very little overlap has been reported between the gene expression profiles of mutant strains *swi4Δ* and *slt2Δ* upon heat shock. Genes dependent on Swi4 but independent on both Swi6 and Slt2, such as *TPK1* [131]*,* were described [127]. Therefore, the expression of Tpk1 subunit isoform, in addition to cAMP-PKA, is regulated by the CWI pathway in response to heat stress (**Figure 2**).

All this evidence highlights the crosstalk between CWI and PKA pathways. It suggests that the response to thermal stress is achieved through a complex and coordinated mechanism in which the effectors of one of the signalling pathways regulate the specificity of the response of the other pathway, allowing a precise and rapid cellular response to overcome the unfavourable environment.

## EFFECT OF STRESS ON THE SPECIFICITY REGULATION OF THE cAMP-PKA PATHWAY

Different external signals trigger the production of cAMP as the only second messenger in the cAMP-PKA signalling. Considering the multiple functions of this pathway in *S. cerevisiae*, an important question is how this kinase achieves specificity, that is, how the cell accomplishes the accurate substrate phosphorylation in response to different stimuli. The three Tpk isoforms are functionally redundant for cell viability despite each one performing specific functions [100, 101, 132–135]. The specificity of PKA signalling in *S. cerevisiae* is regulated by several mechanisms. Below, we describe these mechanisms, highlighting those related to thermal and osmotic stress.

### PKA anchoring through Bcy1 interacting proteins

Yeast PKA localisation appears to be different from that described for mammals. Bcy1 localisation is variable and responsive to environmental and nutritional conditions [116, 136]. Bcy1 N-terminus structure is similar to the canonical mammal RIIα domain (DD domain) as it has a helix-turn-helix motif and the critical amino acids for dimerization [137, 138]. However, the binding domain of proteins described as Bcy1 interactors in *S. cerevisiae* displays different molecular features than the canonical domain of their mammalian counterparts, AKAPs (A-Kinase Anchoring Proteins, DD-AKAP), which contain essential hydrophobic residues [139–141].

The N-terminal domain of Bcy1 and two clusters of phosphorylated serine residues located at this domain have been reported to be critical for Bcy1 cytoplasmic localisation in cells deprived of glucose [142]. In cells growing exponentially on glucose, a number of proteins that interact with Bcy1 have been identified. Zds1, the first one, participates in the cytoplasmic localisation of Bcy1 [142]. Other Bcy1-interacting proteins as Hsp60 (mitochondrial chaperonin), Eno2 (enolase II), and Ira2 (RAS GTPase-activating protein) were identified [143] (**Figure 3A**). However, no Bcy1-interacting proteins were associated with a role in the heat or osmotic stress response. Further studies of Bcy1-interacting proteins will enhance our understanding of the intricate mechanism governing the role of PKA-anchoring proteins in stress response in yeast.

### Subcellular localisation of Tpk1, Tpk2 and Tpk3 catalytic isoforms in response to stress

In *S. cerevisiae*, the localisation of each PKA subunit in different subcellular compartments and structures is affected by several environmental conditions, such as glucose deprivation, thermal and osmotic stress or quiescent arrest [116, 136] (**Figure 3**). When yeast cells are grown in the presence of glucose, both Bcy1 and Tpk2 are localised in the nucleus; however, Tpk1 and Tpk3 subunits are equally distributed in nucleus and cytoplasm [116] (**Figure 3A**). On the other hand, when yeast cells are grown in the presence of glycerol or when entering in the stationary phase, both Tpks and Bcy1 subunits are localised mainly in cytoplasm [116] (**Figure 3B**).

As mentioned above, Tpk1 accumulates in the nucleus, whereas the localisation of Tpk2 and Bcy1 does not change in response to osmostress [82]. Under osmotic stress, the coding regions of osmo-inducible genes recruit both Tpk1 and Tpk2 subunits, whereas promoter regions of ribosomal protein genes exclusively recruit Tpk2 [82] (**Figure 3B**).

On the other hand, upon heat stress, during glucose starvation or in quiescent cells, Tpk1 and Bcy1 display a diffuse cytoplasmic localisation, while Tpk2 and Tpk3 subunits are assembled in PBs and SGs [112, 116, 144] (**Figure 3B**). The severity of the heat stress also regulates the localisation of PKA subunits. In response to a mild heat stress, Tpk2 localisation is cytoplasmic instead of nuclear, and Tpk3 condensates in cytoplasmic foci that are different to classical SGs or PBs. On the other hand, both Tpk2 and Tpk3 subunits are assembled in SGs under severe heat stress [119]. When the cells are treated with cycloheximide and then subjected to heat stress, the foci containing Tpk2 and Tpk3 are not detected, indicating that these foci are dependent on the translation initiation repression [119]. The granular localisation of Tpk2, but not that of Tpk3, depends on its catalytic activity. All these results suggest that different mechanisms are involved in the assembling of each catalytic subunit in response to severe heat stress [119]. A breakthrough in this topic is the demonstration that the N-terminus of Tpk2 subunit has a prion-like domain necessary to localise this catalytic isoform to PBs and SGs upon heat stress, under glucose depletion and after quiescent arrest [144].

Therefore, this evidence indicates that, in response to stress, each PKA isoform employs a unique mechanism for subcellular localisation, complexing each Tpk with a specific subgroup of putative substrates.

### Transcriptional regulation of PKA subunits

Pioneer high-throughput transcriptomic studies have shown that the expression of *TPK1*, *TPK2*, *TPK3* and *BCY1* genes is upregulated in response to heat shock and saline stress [29, 149–153]. However, later published evidence demonstrated that the expression of each PKA subunit is differentially regulated under different growth conditions such as carbon source availability or growth phase [116, 145]. In addition, PKA activity regulates the transcription of the three catalytic isoforms and Bcy1 subunits that compose the holoenzyme [120].

Tpk2 catalytic subunit shows the highest inhibitory effect on the activity of *TPK1* and *TPK3* promoters but fails to inhibit the *TPK2* promoter [120].

From all the subunits that compose PKA, only the expression of Tpk1 is modulated during heat shock and osmostress. Under these conditions, both mRNA levels and half-life increase. In response to heat shock, the upregulation of *TPK1* depends on the transcription factors Msn2/4, Gis1, Sok2, and the kinase Rim15. During the *TPK1* promoter activation, three positioned nucleosomes are evicted [120, 146]. The chromatin remodelling involves the activity of the remodelers RSC and INO80 to maintain the repression of *TPK1* promoter under normal growth conditions, and the complex SWI/SNF to allow the activation after thermal stress [146]. Msn2/4 are necessary for the recruitment of the SWI/SNF complex. Strikingly, the catalytic subunits Tpk1 and Tpk2 are both recruited to the *TPK1* promoter upon heat shock but with opposite temporal patterns [146]. Tpk1 subunit shows its maximum recruitment at 30 min post-heat stress and a significant decrease after 120 min. On the contrary, Tpk2 subunit recruitment shows a slight decrease from 0 min to 30 min post-stress and a maximum recruitment at 120 min [146]. Furthermore, Tpk1 and Tpk2 catalytic activities have opposite effects on the chromatin remodelling of this promoter [146]. The kinetics of association of Tpk1 subunit at the promoter is consistent with the requirement of its activity for chromatin remodelling and the increments of *TPK1* mRNA levels upon heat stress. The authors hypothesize that the recruitment of Tpk2 at *TPK1* promoter may collaborate with the turn off of transcription and the shutdown of the signalling, although further studies must be carried out to fully elucidate this hypothesis [146]. Therefore, a complex regulation mechanism involves the activity of Tpk subunits on the *TPK1* promoter.

Finally, after thermal stress, the increased level of Tpk1 allows the formation of PKA holoenzymes containing a higher proportion of the catalytic Tpk1 isoform. This holoenzyme might phosphorylate Tpk1 specific substrates improving the overall cellular fitness when normal environmental conditions are restored [131].

Thus, the results uncover a particular mechanism involved in the regulation of Tpk1 subunit expression by thermal stress that contributes to defining the specificity of the cAMP-PKA pathway in the response to stress.

## CONCLUSION

To respond adequately to stressors, *S. cerevisiae* cells employ different signalling pathways. Each pathway is fine-tuned through mechanisms that allow the specificity of the response. The complexity of the inputs to which the yeast may be exposed suggests that several pathways should be interconnected to process environmental signals and to achieve a specific response. Two important crosstalk interactions couple the signalling cAMP-PKA and CWI pathways in response to heat shock, and HOG-MAPK and cAMP-PKA pathways upon osmotic stress.

The regulation of the expression of each PKA subunit is one of the important mechanisms that allows signal transduction specificity. In response to heat shock and osmotic stress, *TPK1* is the only gene encoding PKA subunit which is upregulated, and the cAMP-PKA/CWI crosstalk coordinates Tpk1 expression.

The interaction between HOG-MAPK and cAMP-PKA pathways highlights the differential roles of the catalytic isoforms of PKA, Tpk1 and Tpk2, in the adaptive response to osmotic stress. The deletion of *TPK2* gene, but not *TPK1*, improves the defective cell growth of *HOG1* deficient strains under osmotic stress. PKA catalytic subunits Tpk2 and, to a lesser extent, Tpk1, can reduce the crosstalk between the pMAPK and the HOG-MAPK pathways in a deficient *HOG1* strain. The cAMP-PKA pathway activation is required for filamentous growth and each catalytic isoform has a different role in this process. The invasive growth of a *hog1Δ* strain under hyperosmotic conditions is regulated by a crosstalk between the HOG1-MAPK and fgMAPK pathways. Tpk1 is a positive regulator in this crosstalk, while Tpk2 is a negative one. Finally, there is also an interaction between PKA and *HOG1* at the level of transcriptional regulation of osmostress-responsive genes. *TPK2* and *HOG1* have a reciprocal impact on the chromatin-binding kinetics of Tpk2 and Hog1. Also, both kinases regulate the binding of the SWI/SNF complex and Msn2 to the promoters of osmostress-responsive genes.

In conclusion, intricate regulatory networks that include the crosstalk between different signalling pathways take place in response to stress. The complementation of signalling pathways, the fine tuning of the signals, and the specificity in the response to different stressors are key to produce a precise and timely gene expression output to overcome the stressful conditions.

Gaining insight into the intricate mechanisms governing responses and adaptations to environmental challenges, such as heat and hyperosmotic stress, is crucial for optimizing key industrial processes. The identified and comprehended interactions serve as a foundation for improving the efficiency and robustness of various processes, including fermentation. These precise insights into cellular responses provide valuable information for crafting metabolic engineering strategies and refining process control, proving instrumental for the industry. Ultimately, ongoing advancements in this field will not only deepen our comprehension of cellular biology but also drive practical innovations that significantly enhance industrial production.

## AUTHOR CONTRIBUTION

Conceptualization, P.P and S.R; review and editing, M.C.O-M, F.G. and M.B-M; supervision, P.P. and S.R.; project administration, P.P and S.R.; funding acquisition, P.P and S.R. All authors have read and agreed to the published version of the manuscript.

## Abbreviations:

AKAPs: – A-kinase anchoring proteins.
CWI: – cell wall integrity,
ESR: – environmental stress response,
HOG-MAPK: – high osmolarity glycerol-mitogen activated protein kinase,
Hsf1: – heat shock factor 1,
HSR: – heat shock response,
PB: – processing bodies,
PKA: – cAMP-protein kinase A,
RBPs: – RNA-binding proteins,
RNP: – ribonucleoprotein,
SG: – stress granules,
